# Advancing implementation science in community settings: the implementation strategies applied in communities (ISAC) compilation

**DOI:** 10.1186/s12966-024-01685-5

**Published:** 2024-11-26

**Authors:** Laura E. Balis, Bailey Houghtaling, Whitney Clausen, Hannah Lane, Marilyn E. Wende, Emiliane Pereira, Gabriella M. McLoughlin, Samantha M. Harden

**Affiliations:** 1 Center for Nutrition & Health Impact, Omaha, NE USA; 2 Human Nutrition, Foods, and Exercise, Virginia Tech, Blacksburg, VA USA; 3 Duke University School of Medicine, Durham, NC USA; 4 Department of Health Education and Behavior, University of Florida, Gainesville, FL USA; 5 Department of Health Promotion, University of Nebraska Medical Center, Omaha, NE USA; 6 Department of Social and Behavioral Sciences, Temple University College of Public Health, Philadelphia, PA USA; 7 Washington University Implementation Science Center for Cancer Control (WUISC3), St. Louis, MO USA

**Keywords:** Implementation strategies, Community settings, Contextual factors, Pragmaticism, Primary prevention, Public health

## Abstract

**Background:**

Implementation strategies have predominantly been operationalized and studied in clinical settings. Implementation strategies are also needed to improve evidence-based intervention (EBI) integration in community settings, but there is a lack of systematic characterization of their use, which limits generalizability of findings. The goals of this study were to determine which implementation strategies are most used to deliver primary prevention EBIs in community settings, develop a compilation and pragmatic strategy selection process with accompanying guidance tools, and understand practitioners’ preferences for dissemination.

**Methods:**

Purposive and snowball sampling was used to recruit community setting researchers and practitioners delivering primary prevention EBIs (nutrition, physical activity, tobacco prevention) in community settings: education, social services, city planning and transportation, workplaces, recreation/sport, faith-based, and other public health organizations. Semi-structured interviews were conducted using a guide based on the reach, effectiveness, adoption, implementation, maintenance (RE-AIM) framework. Participants were asked to describe barriers experienced and strategies used to overcome them within each RE-AIM dimension. Practitioners were also asked about preferred dissemination strategies, prompted by Diffusion of Innovations theory concepts of sources (who provides information) and channels (how information is provided). A rapid deductive approach was used to analyze findings with a coding matrix aligned with the interview guide.

**Results:**

Researchers (*n* = 10) and practitioners (*n* = 8) across all targeted settings and intervention outcomes completed interviews. Interviewees shared unique implementation strategies (*N* = 40) which were used to overcome barriers related to multiple RE-AIM dimensions, most commonly implementation (*n* = 29) and adoption (*n* = 27). Most frequently mentioned implementation strategies were conduct pragmatic evaluation (*n* = 31), provide training (*n* = 26), change adaptable program components (*n* = 26), and leverage funding sources (*n* = 21). Webinars (*n* = 6) and listservs/newsletters (*n* = 5) were the most mentioned dissemination channels; national public health organizations (*n* = 13) were the most mentioned sources.

**Conclusions:**

Results reflect commonly used implementation strategies in community settings (e.g., training, technical assistance) and add novel strategies not reflected in current taxonomies. Dissemination preferences suggest the need to involve broad-reaching public health organizations. The resultant compilation (Implementation Strategies Applied in Communities) and strategy selection process provide resources to assist researchers and practitioners in applying strategies and improving EBI delivery in community settings.

**Supplementary Information:**

The online version contains supplementary material available at 10.1186/s12966-024-01685-5.

**Table:** 

**Text box 1. Contributions to the literature**
• Implementation strategies have primarily been operationalized and studied in clinical settings. Researchers and practitioners in community settings require relevant implementation strategies to integrate evidence-based primary prevention interventions.
• A new compilation developed through this study, ISAC (Implementation Strategies Applied in Communities), includes 16 novel strategies not reflected in current implementation strategy taxonomies (e.g., related to developing pragmatic, adaptable interventions and evaluation methods and using community-engaged processes).
• Based on existing literature and the results of the study, a four-step process with accompanying guidance tools was developed for selecting ISAC strategies to address implementation determinants and improve implementation outcomes.

## Background

Translating evidence based interventions (EBIs) into real world settings is challenging: only an estimated 14% of original research is delivered as intended, and this process takes 15–17 years on average [1–3]. As a result, many EBIs are not reaching community members who could benefit from them. These issues are well recognized in implementation science [4–6]. However, implementation science methods, models, and measures are predominantly grounded and applied in clinical settings [7–11]. While translating EBIs into practice is challenging in clinical settings, community (non-clinical) settings face distinct challenges that may require specific methods, models and measures [12–14].

Research to practice challenges also hinder achieving health equity (i.e., opportunities for all to achieve optimal health) [15–19]. Barriers to integrating EBIs can be exacerbated in settings with lower resources, which often reach community members facing health disparities [13, 16, 20]. For example, adoption may be affected by competing demands and burdensome programs; implementation may be impacted by EBIs that are considered a poor fit or by a lack of implementation support; reach may be lower due to participation barriers (e.g., work schedules or limited transportation) and limited culturally appropriate promotion; and maintenance can be difficult due to lower resources and capacity among community settings [21]. When differences between settings are not addressed, health outcomes may improve in higher-resource but not lower-resource settings, leading to inequalities generated by EBIs [22–24].

Taken together, to improve overall public health impact in community settings and avoid exacerbating health disparities, there is a need to improve reach, adoption, implementation, and maintenance of EBIs – especially in settings with lower resources. The field of implementation science offers opportunities for researchers and practitioners to improve implementation outcomes including adoption, implementation, and maintenance through use of implementation strategies, defined as active methods or techniques to move research to practice [25]. Researchers have developed a strong foundation of implementation strategies, with multiple compilations to choose from, such as the Expert Recommendations for Implementing Change (ERIC) compilation [10], Effective Practice and Organization of Care [26], and Leeman et al.’s classes of implementation strategies [27]. While these compilations have created valuable contributions to the field, challenges remain: most compilations originated in clinical healthcare settings and use clinical terminology [10, 26, 27]; others have been adapted for single settings and services (e.g., youth behavioral health) [28]. Overall, existing compilations are not easily applied to diverse community settings [12].

Community settings are organizations that deliver public health EBIs but have missions beyond health care [13]. They include education, social services, city planning and transportation, workplaces, recreation/sport, faith-based [13], and other non-clinical public health settings (e.g., the national Cooperative Extension System and non-clinical services provided by public health departments) [12, 13]. Community settings deliver programs in settings where people live, learn, work, and play [29, 30]. In the field of chronic disease prevention, EBIs delivered in these settings are typically primary prevention interventions [13], directed at modifiable risk factors (e.g., dietary quality, physical activity, tobacco use) [13, 31]. These EBIs are increasingly delivered at the policy, systems, or environmental levels (vs. individual or interpersonal levels) [32, 33] to address social determinants of health [23, 29, 34].

Considering the diverse community settings EBIs are implemented in and the multi-level, population-focused nature of the EBIs, it may be more difficult to integrate EBIs in community than clinical settings. While there are determinants at many levels that affect EBI integration, the inner setting [35] in particular is different between clinical and community settings. First, community settings often have missions, cultures, capacity, and resources that are not focused on public health [13]. For example, the primary focus may be on youth development, education, or profit (e.g., retail settings) [36–38]. Second, community settings often do not have consistent resources or funding to support public health EBIs [13, 35]. Third, community settings may lack access to health-related data [13], and evaluating intervention effectiveness is often a challenge [39–41]. Fourth, the success of interventions in community settings may rely on recruiting the priority population to participate (e.g., in health promotion programs) [39, 42] compared to clinical setting interventions that reach existing patient populations. Thus, there is a need for relevant implementation strategies to support community setting researchers and practitioners in overcoming these barriers.

While implementation strategies are needed to improve EBI integration in community settings, there is a lack of systematic characterization of their use. This may be a result of perceived limited applicability, lack of relevant language, and/or dissemination challenges for existing compilations [12–14]. When practitioners and researchers in community settings use implementation strategies, they are often not described with terminology that matches available compilations [12, 14]. Based on implementation strategies used in community settings that *are* specified by name, the scope of use appears to be limited [12]. Practitioners and researchers in community settings often report using “training and hoping” [43] and other implementation strategies addressing individual level-barriers, such as educational meetings and materials or outreach visits, instead of using the full spectrum of available implementation strategies to address barriers at organizational in addition to individual levels [14].

These challenges have slowed the advancement of implementation science in community settings and resulted in a lack of information on which implementation strategies are used in community settings and their impact on EBI delivery [12, 14]. To address these gaps, previous work introduced community setting researchers and practitioners to implementation strategies by providing terminology and examples [12]. This included language tailored from the ERIC compilation and provided relevant community setting examples [12]. For instance, “patients” was changed to “participants” or “priority population” and “clinical innovation” was changed to “evidence-based intervention” [12]. However, questions remain related to which implementation strategies are relevant in community settings and which may not have been captured through tailoring a clinical setting compilation.

In addition, there is a need to provide guidance to researchers and practitioners in community settings on selecting relevant implementation strategies to overcome barriers and capitalize on facilitators. Existing implementation strategy selection methods (e.g., concept mapping, group model building, conjoint analysis, implementation mapping, and causal pathway development) can be complex and costly, and are limited in the extent to which they are used in community settings [44–47]. A simplified approach is needed for community settings to engage in the implementation strategy selection process without dedicated staffing or time [48]. For instance, a pragmatic process with a guidance tool for community settings could be a useful starting point for considering implementation determinants or outcomes and matching implementation strategies [49]. To increase practitioners’ use of a pragmatic compilation and guidance tool, there is a need to understand how to disseminate this work in open-access, community-friendly methods [50]. Given these considerations, the goals of this study were to (1) determine which implementation strategies are commonly used in community settings delivering primary prevention EBIs, and which implementation determinants and outcomes they are used to address, (2) develop a compilation of Implementation Strategies Applied in Communities (ISAC) and a pragmatic strategy selection process with accompanying guidance tools based on this information, and (3) understand practitioners’ preferences for learning about the compilation.

## Methods

### Study design

The study employed an emic (i.e., focusing on the “insider’s perspectives”), qualitative design following an adapted freelisting approach [51, 52]. That is, instead of beginning with and adapting an existing implementation strategy compilation, the goal was to understand implementation barriers and strategies used to overcome them in participants’ own words. This research was deemed exempt by the University of Nebraska Medical Center IRB, #0257-23-EX.

### Participants and recruitment

Purposive and snowball sampling [53, 54] were used to recruit public health researchers and practitioners operating across seven types of community settings: education, social services, city planning and transportation, workplaces, recreation/sport, faith-based, and other non-clinical public health settings [13]. Purposive sampling was used to identify researchers and practitioners through the lead author’s (LB) previous experience in community-based implementation science and by engaging with the Community Participation Action Group of the Consortium for Cancer Implementation Science (CCIS) [55, 56]. Snowball sampling was conducted by asking researcher interviewees for recommendations of community partners to interview.

To engage with prospective participants, up to three recruitment emails (Supplemental File 1) were sent including the purpose of the research and a link to a screening survey (Supplemental Files 2 and 3) regarding participation eligibility. To be eligible, researchers had to have experience conducting community-engaged research to improve the adoption, implementation, or maintenance of an EBI in physical activity, nutrition, or tobacco prevention (primary prevention for chronic diseases) [57–59] in non-clinical settings. For practitioners, the eligibility requirement was centered on experience managing or coordinating these types of EBIs. The screening survey also queried data pertaining to (1) which of the noted settings respondents conducted research/worked in, (2) years of research/work experience, (3) and examples of up to three EBIs respondents had implemented, including the intervention level (individual or interpersonal and/or policy, systems, or environmental) [32, 60] and primary outcomes (physical activity, nutrition, or tobacco use). Researchers also self-reported their expertise level in implementation science (beginner, intermediate, advanced) [61]; practitioners were not asked this question.

Recruitment emails were sent to 23 researchers and 37 practitioners (*n* = 60). Of these potential participants, 10 researchers and eight practitioners (*n* = 18) completed the screening survey and participated in an interview. Those who did not participate either did not complete the screening survey (*n* = 33) (two provided reasons, noting time and employer constraints), could not be reached to due to undeliverable email addresses (*n* = 6), or completed the screening survey but were ineligible to participate (*n* = 2) because they answered “no” to the eligibility question (“Through your community-engaged research, have you worked to improve the adoption, implementation, or maintenance of an evidence-based intervention aimed at improving physical activity, nutrition, or tobacco patterns/practices?”). Three of the 60 potential interviewees requested a colleague be recruited in their place; two of these contacts subsequently completed interviews.

### Data collection

Semi-structured interviews were conducted and recorded using Zoom from May to August 2023. Interviews lasted an average of 42 min (range of 25–58 min). All interviewers were female with a PhD or master’s degree and experienced in qualitative research and the RE-AIM (reach, effectiveness, adoption, implementation, maintenance) framework [62]. At the time of the study, LB was a Research Scientist, WC was a Project Manager, HL was a Medical Instructor, MW was a Postdoctoral Fellow, and EP was a Graduate Research Assistant. Participants received a $25 gift card as compensation.

Researcher and practitioner interview guides (Supplemental Files 4 and 5) were based on the RE-AIM framework [62]. Participants were first asked to describe the EBIs they have implemented, then were guided through each RE-AIM dimension and asked what barriers occurred during associated research or practice approaches and what strategies were used to overcome barriers. Practitioner interviewees were also asked about preferred dissemination methods for the resultant implementation strategy compilation following Diffusion of Innovations theory [63], regarding sources (who should provide the information) and channels (how should the information be provided). A knowledgeable public health and practice expert was engaged to pilot test and refine interview language before data collection.

### Analysis

Microsoft Excel was used for descriptive statistics including characteristics of the participants and the EBIs captured from the screening survey. A rapid deductive analysis approach was used to analyze semi-structured interview findings [64–66]. To facilitate this process, a template was developed for structured note-taking according to the interview guide, including RE-AIM dimensions, barriers addressed, descriptions of the implementation strategies used to overcome barriers, and a brief descriptive implementation strategy “code” [67] to aid in comparisons across interviews. Another template was developed to capture practitioners’ dissemination preferences and descriptions, which included dissemination strategies and dissemination strategy codes. Two researchers independently listened to one interview and noted findings using the template to test and refine prior to moving forward with data analysis. Audit trails were maintained for all steps of the analysis process [68].

Interviewers completed the rapid analysis template during or after each interview to ensure all information relevant to the analysis was captured. Interviewers also recommended strategy language by completing the implementation strategy code and dissemination strategy code columns. Then, a second researcher (LB, WC, HL, or GMM) listened to interview recordings and made revisions or additions to the interview notes to ensure agreement and finalize the data. Next, two researchers (LB and WC) performed content analysis [67, 69] by collapsing and sorting these “codes” to condense similarly worded implementation strategies (e.g., “train implementers” and “provide training to implementers”) and to categorize dissemination strategies by recommended sources or channels. One researcher (LB) reviewed the collapsed list of strategies and wrote definitions based on the noted descriptions and another (BH) reviewed strategy names and definitions for clarity and suggested revisions, leading to a final list.

To determine when data saturation was reached, the research team adapted the “new information threshold” method, which assesses saturation during analysis by calculating the point when no to little information is acquired from additional data [70]. This is accomplished through calculating the amount of information present in a set of initial data (the “base”) and in sets of subsequently acquired data (the “run”). The new information threshold is calculated by dividing the amount of new information in each run by the amount of information in the base. The first step in completing this process is to prospectively determine the base size, run length, and new information threshold. Base sizes are recommended as four to six data collection events, as most information is generated early in the qualitative research process, and run length are recommended as two to three. For this study, the research team selected a higher base size (six interviews, three each researchers and practitioners) and run length (three interviews) as the population was diverse in terms of roles and settings. The new information threshold was set at *≤* 5%. New implementation strategies identified in a run were divided by number of implementation strategies present in the base until the ratio was *≤* 5%. Saturation was reached after 15 interviews, and the remaining scheduled interviews were completed.

Next, additional steps were taken to compare the resulting ISAC compilation strategies with ERIC implementation strategies to determine similarities. One researcher (LB) reviewed the final list of ISAC implementation strategies, searched for ISAC implementation strategy terms and synonyms in the ERIC compilation (strategy names and definitions), and reviewed all remaining ERIC strategies. ERIC strategies with similar content (but different language) were linked to the most relevant ISAC strategy. A second researcher (BH) reviewed both compilations and the list of similar strategies, noted areas of disagreement, and reconciled through discussion and agreement with LB.

Finally, data were analyzed to develop two guidance tools for selecting implementation strategies. Through the CCIS Action Group, researchers and practitioners expressed a preference for tools based on two commonly used frameworks: RE-AIM [62] and the Consolidated Framework for Implementation Research (CFIR) [35]. The pragmatic RE-AIM guidance tool was designed to provide guidance on which RE-AIM dimension (implementation outcome) each implementation strategy was used to address. It was developed by calculating the frequency of RE-AIM dimensions mentioned for each final implementation strategy. RE-AIM dimensions were indicated in the tool as relevant to each implementation strategy if they were mentioned at least two times or mentioned once for implementation strategies with frequency of *≤* 3 mentions.

To ensure broad applicability across determinant frameworks, the second tool was developed as a guidance tool aligning with CFIR and other determinant frameworks (e.g., Practical, Robust Implementation and Sustainability Model [71]; Integrated-Promoting Action on Research Implementation in Health Services [72, 73]; and Exploration, Preparation, Implementation, Sustainment [74]). It was designed to provide guidance on which determinant each implementation strategy was used to overcome. Additional coding was completed to assess which CFIR domain (individuals, innovation, inner setting, outer setting, and implementation process) described each barrier resolved by an implementation strategy. A research assistant with experience supporting implementation science investigations in community settings reviewed the final list and applied codes. A second researcher (WC) reviewed these codes and indicated inconsistencies; the lead researcher (LB) reviewed and reconciled. The guidance tool was developed by calculating the frequency of determinant framework domains that were resolved by each final implementation strategy. CFIR domains were indicated in the tool as relevant to each implementation strategy if they were mentioned at least two times or mentioned once for implementation strategies with frequency of *≤* 3 mentions.

## Results

### Interviewee and EBI characteristics

Ten researchers (56%) and eight practitioners (44%) across all seven targeted community settings and three intervention outcomes completed interviews (*n* = 18). Researchers had on average 9.4 (SD *±* 6.40) years of experience, and half rated themselves as intermediate-level implementation scientists (*n* = 5, 50%). Practitioners had 15.8 (SD *±* 5.97) years of experience on average. Based on information from organizational websites, participants were located in all United States census regions, with most (*n* = 6 researchers, 60%, and *n* = 4 practitioners, 50%) in the southern region.

On the screening survey question prompting respondents to list up to three EBIs delivered, researchers listed an average of 2.0 (SD *±* 0.83) and practitioners listed an average of 2.8 (SD *±* 0.66). All interviewees, including one practitioner interviewee who was suggested by a researcher colleague, listed unique EBIs which were subsequently discussed in interviews. Practitioners reported delivering EBIs across both levels (individual or interpersonal; policy, systems, or environmental levels) more frequently than researchers; both researchers and practitioners often focused on multiple public health outcomes. Half (*n* = 9, 50%) of the interviewees responded that they conduct community-engaged research in (researchers) or work in (practitioners) multiple settings, for a total of 19 research and 35 practice settings. See Table 1 for characteristics of interviewees and EBIs.

**Table 1:** Researcher and practitioner interviewees’ demographic and evidence-based intervention characteristics

Variable	Researchers (*n* = 10)	Practitioners (*n* = 8)
**Years of Experience**,** M (SD)**	9.4 (*±* 6.40)	15.8 (*±* 5.97)
**Implementation Science Expertise**,** n (%)**		
Beginner	2 (20)	n/a
Intermediate	5 (50)	
Advanced	3 (30)	
**Geographic Region (US Census)**,** n (%)**		
Midwest	2 (20)	2 (25)
Northeast	1 (10)	1 (12)
South	6 (60)	4 (50)
West	1 (10)	1 (12)
**Number of Interventions Reported**,** M (SD)**	2.0 (*+ 0.83)*	2.75 (*+ 0.66)*
**Intervention Levels**,** n (%)**		
Individual or interpersonal	4 (40)	1 (12)
Policy, systems, or environmental	3 (30)	0 (0)
Both levels	3 (30)	7 (88)
**Intervention Outcomes**,** n (%)**		
Physical Activity	3 (30)	0 (0)
Tobacco	1 (10)	0 (0)
Nutrition	0 (0)	2 (25)
Multiple outcomes	6 (60)	6 (75)
**Intervention Settings**,** n (%)**	**Research settings (** *n* ** = 19)**	**Work settings (** *n* ** = 35)**
Education	7 (37)	10 (29)
Faith-Based	3 (16)	5 (14)
Recreation/Sport	2 (11)	3 (9)
Social Services	1 (5)	2 (6)
City Planning & Transportation	1 (5)	2 (6)
Workplaces	1 (5)	2 (6)
Other Public Health	4 (21)	11 (31)

### Implementation strategies applied in communities

Across all interviews, implementation strategies were mentioned a total of 432 times, with an average of 24 mentions per interviewee. Following the rapid deductive analysis approach, a total of 40 unique ISAC strategies remained (Table 2). ISAC strategies reported the most across interviewees were conduct pragmatic evaluation (*n* = 31), provide training (*n* = 26), change adaptable program components (*n* = 26), leverage funding sources (*n* = 21), structure grant requirements (*n* = 19), and tailor recruitment strategies (*n* = 19). See Table 2 for the full list of implementation strategies, definitions, and examples. Twenty-four ISAC strategies (60%) were similar to 37 of the 73 ERIC implementation strategies (51%); see Table 3.

**Table 2:** Implementation strategies Applied in communities (ISAC), with definitions and examples

Strategy	Mentions (*n*)	Definition	Examples
Conduct pragmatic evaluation	31	Evaluate success through methods that fit the evidence-based intervention (EBI), personnel, and setting	• Used validated simplified instruments • Shorten survey to maximize the amount of time people are spending in the EBIs • Don’t evaluate “all of the things” to show EBI effectiveness • Collect data that’s important to community partners
Provide training	26	Train staff, volunteers, or partner organizations on the EBI	• Hold booster trainings to help implementers adhere to curriculum • Offer web-based training that can be used to house materials and send reminders • Train implementers on community engagement and diversity, equity, and inclusion • Provide policy training to teach organizations how to better advocate at policy levels for their organizations and EBIs
Change adaptable program components	26	Tailor the program through fidelity-consistent changes to meet the needs of the priority population or setting	• Change physical activities to accommodate the weather and time of day • Use virtual instead of in-person programming when it best meets participants’ needs • Work with the intervention developer to reduce the number of weeks and session time • Reduce elements of complex tobacco policy from 13 to three
Leverage funding sources	23	Access new funding, use creative or untraditional funding sources, or combine funding sources	• Connect communities to other partners that have grants and funding (e.g., department of transportation) • Find funding for one portion of the priority population (e.g., kids in an intergenerational program) to match funding availability and interests • Write a planning grant • Use supplemental COVID funding to do additional outreach and become better integrated in the community
Structure grant requirements	19	Develop requests for applications that prioritize EBIs with high reach to priority populations	• Create funding applications that are friendlier to rural areas with less resources • Designate grantee funding eligibility depending on the EBI’s potential reach • Identify priority populations through the funding application process (e.g., by listing eligible counties) • Work continuously with a core set of grantees to help with continuity
Tailor recruitment strategies	19	Use and adapt multiple recruitment methods to align with the priority populations’ language, culture, or preferences	• Use postcard mailers for recruitment of populations who often do not have cell phones or computers • Have a community contact to ensure that language and recruitment strategies make sense for the priority population • Find out what participants want out of the program, and advertise those benefits (e.g., social vs. physical activity benefits for older adults) • Translate materials into multiple languages
Provide technical assistance	17	Offer guidance (including through external organizations) on implementing an EBI	• Use an external organization to help with scientific background and evaluation • Bring in an external technical assistance provider to hold a Walkability Institute • Connect grantees with EBI-specific technical assistance providers • Hold monthly technical assistance calls to troubleshoot challenges with core components
Change delivery agent roles	15	Alter roles and responsibilities of staff and volunteers to enhance EBI fit and support implementation	• Find alternate roles for volunteers to support the program from behind the scenes • Use community health workers to support the work and recruit participants • Shift practitioners’ focus from direct education to supporting policy, systems, and environmental changes • Allow middle managers to choose which EBIs to implement and evaluate
Leverage program champions	14	Identify and prepare staff and volunteers with high readiness (e.g., influence and attitude)	• Have a community member ‘host’ an event and invite community members to improve recruitment • Identify staff who are passionate about the EBI and can take on more responsibility • Rely on a program champion to initiate activity breaks during meetings • Provide materials and information to guide churches in choosing the right program champion
Choose strategic partner organizations	14	Partner with organizations that have complementary expertise to share resources	• Partner with an organization that excels in training and engaging volunteers • Look for community partners who may be better equipped to deliver programs • Partner with organizations that can offer insurance (if the implementing organization) is unable to do so • Partner with National Heritage Sites that are also interested in implementing Complete Streets
Provide resources	14	Develop and share resources to enhance program delivery	• Provide expert recommendations on child feeding practices • Develop a website with all program resources
Change program delivery site	14	Move programs to different locations or partner organization sites to improve EBI fit or reach	• Implement EBIs in a new area with fewer similar EBIs • Choose a new location based on income, race, or ethnicity to better reach the priority population
Develop a coalition or workgroup	13	Build diverse groups, including partner organizations, community members, and elected officials	• Develop groups of community based organizations representing diverse populations who often don’t have a voice • Develop local coalitions that look like the priority population
Facilitate implementation	13	Offer assistance on the implementation process through an interactive, supportive relationship	• Hold frequent formal and informal conversations with delivery agents to determine what’s working and what’s not • Maintain an ongoing, close working relationship with grantees to help with their needs (e.g., program evaluation)
Design pragmatic programs	12	Develop programs that are low cost, easy to implement, and relevant to multiple populations	• Design simple interventions to avoid fidelity issues • Develop programs that are free and/or use existing resources
Assist with dissemination	12	Offer coaching, mentoring, or other support to aid in sharing results through multiple channels	• Develop a strategic communications and marketing plan • Hold a virtual writing workshop including how to submit manuscripts and go through peer review process, write brief reports, and incorporate visual elements
Use technology for evaluation	11	Use or develop apps, websites, or databases to facilitate improve data collection and sharing	• Use online platform to reduce data issues • Build website to show local elected officials the EBI’s impact on economic indicators
Change programming focus	10	Collaboratively prioritize EBIs that align with priorities and can demonstrate impact	• Change priorities to implement programs that can be evaluated and show impact • Implement policy, systems, or environment-level interventions for longer-lasting impact
Conduct fidelity checks	9	Measure fidelity to assess whether the EBI is being implemented as designed	• Conduct in-person site visits • Assess fidelity (whether core components were done) in program evaluation
Meet community partners’ needs	9	Select or adapt programs to respond to community partners’ needs, concerns, or resources	• Get buy-in from the community and work to meet community partners’ objectives • Meet communities where they are at, and start from there to implement feasible EBIs
Connect practitioners with experts	9	Facilitate external connections to help with program implementation or evaluation	• Connect communities with universities, researchers, or students to get help with evaluation metrics • At the outset, sit down with experts ask what program components can’t be changed
Assist with selecting EBIs	8	Provide options and assistance for choosing the best-fit EBIs	• Offer a menu of interventions for implementers to choose from • Help grantees select interventions that are needed and a good fit
Conduct needs assessment	8	Use qualitative or quantitative methods to understand needs of the priority population and EBI implementers	• Hold focus groups with residents to understand views of built environment and important destinations that could be linked • Conduct survey with staff, administrators, and parents asking about needs, interest, and how can they be supported
Engage potential partners	8	Use networking, relationships, or outreach events to share information about the EBI with potential partners	• Present at statewide meetings to gain buy-in • Find schools that may be willing to participate through personal, informal networks
Enhance staffing	8	Consider a staffing plan that facilitates high-quality, consistent delivery of the EBI.	• Hire at a local level (people who represent the priority population) to work on the EBI • Use a project manager to coordinate among multiple organizations working together to deliver the EBI
Meet participants’ needs	6	Address participants’ needs to increase engagement	• Making programming engaging and learner-centered • Ensure participants have transportation
Create program guide	6	Create a playbook, blueprint, or guide describing how to implement the EBI	• Build and maintain protocols to hand off to new staff • Create a blueprint to give leaders more information upfront on what to expect
Use formal agreements with partners	6	Establish memorandums of understanding (MOUs), policies, or other agreements to share space and ensure supports are available.	• Create a commitment form for leaders who are essential to program success. • Develop an MOU with after school programs to ensure staff are available during EBI delivery
Use reimbursement systems	6	Advocate for or establish systems for payors (insurers, employers) to reimburse EBI costs	• Help worksites to explore insurance coverage or worksite benefit • Advocate for legislation to get Medicaid to reimburse for the EBI
Engage community members	6	Share information about the EBI and seek community member input	• Create a survey for community members to give input on the proposed EBI • Ask for input on adaptable program components (e.g., music, language, images) to enhance cultural appropriateness
Plan for sustainability	5	Set up funding structures to support program maintenance, and work with implementing sites to provide resources and support to continue the EBI	• At the conclusion of the EBIs, work with faith-based organizations to develop a plan for the next year • Continue institutional support by providing resources
Build partner relationships	5	Invest time to build long-term relationships with community partners	• Hold multiple conversations or meetings to build trust and long-term relationships • Come in to partnerships with an open mind, not a set agenda
Train the trainer	5	Train implementers or volunteers to train others to implement EBIs.	• Train master trainers who train coordinators who train volunteers • Train Master Wellness Volunteers to do content education and technical assistance
Facilitate peer learning	5	Set up networking and collaborating opportunities for implementers to learn from peers	• Serve as a convenor to help organizations learn from each other • Use a peer learning collaborative model, including monthly reflection prompts and goal setting
Reassess inclusion criteria	4	Promote broad reach by reducing exclusion criteria	• Lift inclusion requirements as programs are scaled • Reduce EBI exclusion criteria (compared to other EBIs) to increase participation
Review performance and provide feedback	4	Review performance or evaluation results and provide feedback to facilitate improvements	• Use audit and feedback to evaluate teachers against their own goals • Report state-level feedback so missing data points are easy to see
Develop structured curriculum	4	Develop structured curriculum, including lessons plans and other content, for program implementers	• Include recipes that align with nutrition guidelines and the EBI curriculum • Develop lesson plans and other content for internal staff and other program implementers
Develop adaptable programs	3	Ensure programs are adaptable to meet the needs of organizations and priority populations	• Develop programming as a package so worksites can choose what they want to implement given employees’ needs • Create adaptable EBIs so implementers can make changes based on community and staffing needs
Incentivize delivery agents	3	Incentivize program implementers through payment structures or other rewards	• Pay trainers to be certified and deliver program sessions • Offer credits to students who volunteer
Conduct demonstration events	2	Short-term environmental changes to assess use and gather community member feedback	• Host a temporary “pop-up” demonstration event to get feedback on proposed environmental changes

**Table 3:** Comparison of implementation strategies in the ISAC compilation to the ERIC compilation

ISAC strategy and definition	Similar ERIC strategies and definitions
**Assist with dissemination**: Offer coaching, mentoring, or other support to aid in sharing results through multiple channels	None
**Assist with selecting EBIs**: Provide options and assistance for choosing the best-fit EBIs	None
**Build partner relationships**: Invest time to build long-term relationships with community partners	None
**Change adaptable program components**: Tailor the program through fidelity-consistent changes to meet the needs of the priority population or setting	**Promote adaptability**: Identify the ways a clinical innovation can be tailored to meet local needs and clarify which elements of the innovation must be maintained to preserve fidelity
**Change delivery agent roles**: Alter roles and responsibilities of staff and volunteers to enhance EBI fit and support implementation	**Revise professional roles**: Shift and revise roles among professionals who provide care, and redesign job characteristics
**Change program delivery site**: Move programs to different locations or partner organization sites to improve EBI fit or reach	**Change service sites**: Change the location of clinical service sites to increase access
**Change programming focus**: Collaboratively prioritize EBIs that align with priorities and can demonstrate impact	None
**Choose strategic partner organizations**: Partner with organizations that have complementary expertise to share resources	None
**Conduct demonstration events**: Short-term environmental changes to assess use and gather community member feedback	**Model and simulate change**: Model or simulate the change that will be implemented prior to implementation.
**Conduct fidelity checks**: Measure fidelity to assess whether the EBI is being implemented as designed	**Develop and implement tools for quality monitoring**: Develop, test, and introduce into quality-monitoring systems the right input—the appropriate language, protocols, algorithms, standards, and measures (of processes, patient/consumer outcomes, and implementation outcomes) that are often specific to the innovation being implemented. **Develop and organize quality monitoring systems**: Develop, test, and introduce into quality-monitoring systems the right input—the appropriate language, protocols, algorithms, standards, and measures (of processes, patient/consumer outcomes, and implementation outcomes) that are often specific to the innovation being implemented.
**Conduct needs assessment**: Use qualitative or quantitative methods to understand needs of the priority population and EBI implementers	**Conduct local needs assessment**: Collect and analyze data related to the need for the innovation
**Conduct pragmatic evaluation**: Evaluate success through methods that fit the evidence-based intervention (EBI), personnel, and setting	None
**Connect practitioners with experts**: Facilitate external connections to help with program implementation or evaluation	**Use an implementation advisor**: Seek guidance from experts in implementation **Develop academic partnerships**: Partner with a university or academic unit for the purposes of shared training and bringing research skills to an implementation project **Work with educational institutions**: Encourage educational institutions to train clinicians in the innovation.
**Create program guide**: Create a playbook, blueprint, or guide describing how to implement the EBI	**Develop a formal implementation blueprint**: Develop a formal implementation blueprint that includes all goals and strategies. The blueprint should include: (1) aim/purpose of the implementation; (2) scope of the change (e.g., what organizational units are affected); (3) timeframe and milestones; and (4) appropriate performance/progress measures. Use and update this plan to guide the implementation effort over time.
**Design pragmatic programs**: Develop programs that are low cost, easy to implement, and relevant to multiple populations	None
**Develop a coalition or workgroup**: Build diverse groups, including partner organizations, community members, and elected officials	**Build a coalition**: Recruit and cultivate relationships with partners in the implementation effort **Use advisory boards and workgroups**: Create and engage a formal group of multiple kinds of stakeholders to provide input and advice on implementation efforts and to elicit recommendations for improvements.
**Develop adaptable programs**: Ensure programs are adaptable to meet the needs of organizations and priority populations	None
**Develop structured curriculum**: Develop structured curriculum, including lessons plans and other content, for program implementers	None
**Engage community members**: Share information about the EBI and seek community member input	**Involve patients/consumers and family members**: Engage or include patients/consumers and families in the implementation effort. **Obtain and use patients/consumers and family feedback**: Develop strategies to increase patient/consumer and family feedback on the implementation effort.
**Engage potential partners**: Use networking, relationships, or outreach events to share information about the EBI with potential partners	**Conduct educational outreach visits**: Have a trained person meet with providers in their practice settings to educate providers about the clinical innovation with the intent of changing the provider’s practice.
**Enhance staffing**: Consider a staffing plan that facilitates high-quality, consistent delivery of the EBI	None
**Facilitate implementation**: Offer assistance on the implementation process through an interactive, supportive relationship	**Facilitation**: A process of interactive problem solving and support that occurs in a context of a recognized need for improvement and a supportive interpersonal relationship.
**Facilitate peer learning**: Set up networking and collaborating opportunities for implementers to learn from peers	**Create a learning collaborative**: Facilitate the formation of groups of providers or provider organizations and foster a collaborative learning environment to improve implementation of the clinical innovation. **Capture and share local knowledge**: Capture local knowledge from implementation sites on how implementers and clinicians made something work in their setting and then share it with other sites.
**Incentivize delivery agents**: Incentivize program implementers through payment structures or other rewards	**Alter incentive/allowance structures**: Work to incentivize the adoption and implementation of the clinical innovation.
**Leverage funding sources**: Access new funding, use creative or untraditional funding sources, or combine funding sources	**Access new funding**: Access new or existing money to facilitate the implementation
**Leverage program champions**: Identify and prepare staff and volunteers with high readiness (e.g., influence and attitude)	**Identify and prepare champions**: Identify and prepare individuals who dedicate themselves to supporting, marketing, and driving through an implementation, overcoming indifference or resistance that the intervention may provoke in an organization
**Meet community partners’ needs**: Select or adapt programs to respond to community partners’ needs, concerns, or resources	None
**Meet participants’ needs**: Address participants’ needs to increase engagement	**Intervene with patients/consumers to enhance uptake and adherence**: Develop strategies with patients to encourage and problem solve around adherence.
**Plan for sustainability**: Set up funding structures to support program maintenance, and work with implementing sites to provide resources and support to continue the EBI	None
**Provide technical assistance**: Offer guidance (including through external organizations) on implementing an EBI	**Provide local technical assistance**: Develop and use a system to deliver technical assistance focused on implementation issues using local personnel **Provide ongoing consultation**: provide ongoing consultation with one or more experts in the strategies used to support implementing the innovation **Centralize technical assistance**: Develop and use a centralized system to deliver technical assistance focused on implementation issues.
**Provide training**: Train staff, volunteers, or partner organizations on the EBI	**Conduct ongoing training**: Plan for and conduct training in the clinical innovation in an ongoing way **Make training dynamic**: Vary the information delivery methods to cater to different learning styles and work contexts, and shape the training in the innovation to be interactive
**Provide resources**: Develop and share resources to enhance program delivery	**Develop educational materials**: Develop and format manuals, toolkits, and other supporting materials in ways that make it easier for stakeholders to learn about the innovation and for clinicians to learn how to deliver the clinical innovation. **Distribute educational materials**: Distribute educational materials (including guidelines, manuals and toolkits) in person, by mail, and/or electronically.
**Reassess inclusion criteria**: Promote broad reach by reducing exclusion criteria	None
**Review performance and provide feedback**: Review performance or evaluation results and provide feedback to facilitate improvements	**Audit and provide feedback**: Collect and summarize clinical performance data over a specified time period and give it to clinicians and administrators to monitor, evaluate, and modify provider behavior. **Facilitate relay of clinical data to providers**: Provide as close to real-time data as possible about key measures of process/outcomes using integrated modes/channels of communication in a way that promotes use of the targeted innovation.
**Structure grant requirements**: Develop requests for applications that prioritize EBI with high reach to priority populations	None
**Tailor recruitment strategies**: Use and adapt multiple recruitment methods to align with the priority populations’ language, culture, or preferences	None
**Train the trainer**: Train implementers or volunteers to train others to implement EBIs.	**Use train-the-trainer strategies**: Train designated clinicians or organizations to train others in the clinical innovation.
**Use formal agreements with partners**: Establish memorandums of understanding (MOUs), policies, or other agreements to share space and ensure supports are available.	**Develop resource sharing agreements**: Develop partnerships with organizations that have resources needed to implement the innovation **Obtain formal commitments**: Obtain written commitments from key partners that state what they will do to implement the innovation.
**Use reimbursement systems**: Advocate for or establish systems for payors (insurers, employers) to reimburse EBI costs	**Fund and contract for the clinical innovation**: Governments and other payers of services issue requests for proposals to deliver the innovation, use contracting processes to motivate providers to deliver the clinical innovation, and develop new funding formulas that make it more likely that providers will deliver the innovation. **Place innovation on fee for service lists/formularies**: Work to place the clinical innovation on lists of actions for which providers can be reimbursed (e.g., a drug is placed on a formulary, a procedure is now reimbursable).
**Use technology for evaluation**: Use or develop apps, websites, or databases to facilitate improve data collection and sharing	None

Of the 40 ISAC strategies, most (*n* = 26, 65%) were used to overcome barriers related to multiple RE-AIM dimensions, including implementation (*n* = 29, 73%), adoption (*n* = 27, 67%), reach (*n* = 14, 35%), maintenance (*n* = 13, 33%), and effectiveness (*n* = 7, 18%) (Table 4). Implementation barriers were commonly described in relation to achieving fidelity (*n* = 22), with fewer focused on cost (*n* = 7). As few ISAC strategies were described regarding assessing participants’ long-term outcomes (i.e., the individual-level maintenance dimension), individual-level and organizational-level maintenance barriers were reported together. See Table 4.

**Table 4:** Implementation outcomes (RE-AIM dimensions) influenced by each ISAC strategy

ISAC Strategy	Reach	Effectiveness	Adoption	Implementation - Fidelity	Implementation - Cost	Maintenance
Assist with dissemination						X
Assist with selecting EBIs		X	X	X		
Build partner relationships			X	X		
Change adaptable program components	X		X	X		X
Change delivery agent roles	X		X	X		X
Change program delivery site	X		X	X		
Change programming focus			X		X	X
Choose strategic partner organizations			X		X	
Conduct demonstration events			X			
Conduct fidelity checks				X		
Conduct needs assessment			X			
Conduct pragmatic evaluation		X	X	X		X
Connect practitioners with experts		X		X		
Create program guide				X		
Design pragmatic programs	X		X	X	X	X
Develop a coalition or workgroup	X		X			X
Develop adaptable programs				X		
Develop structured curriculum				X		
Engage community members	X		X			
Engage potential partners			X			
Enhance staffing	X		X		X	
Facilitate implementation		X	X	X		
Facilitate peer learning				X		
Incentivize delivery agents			X			
Leverage funding sources	X		X		X	X
Leverage program champions	X		X			X
Meet community partners’ needs			X			
Meet participants’ needs	X			X		
Plan for sustainability						X
Provide resources	X		X	X		
Provide technical assistance			X	X		
Provide training		X	X	X		X
Reassess inclusion criteria	X					
Review performance and provide feedback				X		
Structure grant requirements	X	X	X		X	X
Tailor recruitment materials	X		X			
Train the trainer				X		
Use formal agreements with partners			X	X		
Use reimbursement systems					X	X
Use technology for evaluation		X	X	X		

*An X denotes that the implementation outcome (RE-AIM dimension) in the column was influenced by the ISAC strategy in the row.

Most of the ISAC strategies (*n* = 28, 70%) were used to overcome barriers related to multiple CFIR domains. Barriers were classified as occurring at level of the outer setting (*n* = 27, 68%), inner setting (*n* = 21, 53%), implementation process (*n* = 21, 53%), innovation (*n* = 12, 53%), and individuals (*n* = 11, 21%). See Table 5 for barriers overcome by determinant level.

**Table 5:** Implementation determinants influenced by each ISAC strategy

ISAC Strategy	Individuals	Innovation	Inner Setting	Outer Setting / Environment	Implementation Process
Assist with dissemination	X				X
Assist with selecting EBIs		X		X	
Build partner relationships			X		X
Change adaptable program components	X	X	X	X	X
Change delivery agent roles		X	X	X	X
Change program delivery site			X	X	X
Change programming focus			X	X	
Choose strategic partner organizations		X	X	X	
Conduct demonstration events				X	
Conduct fidelity checks					X
Conduct needs assessment			X	X	X
Conduct pragmatic evaluation	X		X	X	X
Connect practitioners with experts				X	X
Create program guide			X		
Design pragmatic programs			X		X
Develop a coalition or workgroup			X	X	X
Develop adaptable programs		X		X	X
Develop structured curriculum		X	X		X
Engage community members	X				X
Engage potential partners				X	
Enhance staffing			X	X	
Facilitate implementation				X	X
Facilitate peer learning	X				
Incentivize delivery agents		X		X	
Leverage funding sources			X	X	X
Leverage program champions			X	X	X
Meet community partners’ needs				X	
Meet participants’ needs				X	
Plan for sustainability			X		
Provide resources		X	X		
Provide technical assistance		X	X	X	
Provide training	X	X	X	X	X
Reassess inclusion criteria				X	
Review performance and provide feedback	X				
Structure grant requirements		X	X	X	X
Tailor recruitment materials	X	X	X	X	X
Train the trainer	X				
Use formal agreements with partners	X				
Use reimbursement systems				X	
Use technology for evaluation	X			X	X

*An X denotes that the implementation determinant in the column was influenced by the ISAC strategy in the row.

### ISAC dissemination strategies

Across practitioner interviews, preferred strategies for disseminating ISAC strategies were mentioned a total of 53 times, with an average of seven mentions per interviewee. After collapsing and sorting similar dissemination strategies (e.g., names of specific organizations were collapsed into “Local public health organization” or “National public health organization”), there were 20 unique dissemination source (*n* = 7) and channel (*n* = 13) strategies. National public health organizations in the United States, such as professional associations, accreditors, and non-profit organizations (*n* = 13), and peer learning networks (*n* = 3) were the most mentioned sources for disseminating ISAC strategies. Webinars (*n* = 6), listservs/newsletters (*n* = 5), and conferences (*n* = 3) were the most mentioned dissemination channels.

## Discussion

An emic approach to understanding implementation strategies used by researchers and practitioners in community settings was used to develop ISAC. Given the unique challenges and contextual differences between clinical and community settings, an understanding of common strategies used by community researchers and practitioners was needed [12]. One question in implementation science is how many compilations of implementation strategies are necessary – whether one broad framework should apply to all settings (and be tailored for specific projects) or whether multiple setting-specific compilations are needed. We agree that too many theories, frameworks, and models perpetuates challenges including duplicating efforts and slowing progress. However, we argue that settings with shared language and similar barriers need compilations with familiar terminology and setting-specific implementation strategies. As community-based implementation scientists, we attempted to use ERIC implementation strategies with community partners, but the terminology and strategies did not resonate. Our previous work providing simple terminology changes [12] was inefficient, leading to the conceptualization of the ISAC compilation.

This study uncovered unique implementation strategies (*n* = 16) not included in the ERIC compilation, especially related to developing pragmatic, adaptable interventions and evaluation methods (“Design pragmatic programs”, “Develop adaptable programs”, “Reassess inclusion criteria”, “Conduct pragmatic evaluation”) and using engaged processes to meet the needs of community partners (“Engage potential partners”, “Build partner relationships”, “Meet community partners’ needs”). Further, 36 ERIC strategies were *not* reflected in the ISAC compilation, reinforcing the utility of a separate implementation strategy compilation specific to community settings, while emphasizing some overlap between how EBI adoption, implementation, and maintenance can be supported in community or clinical settings. Given this, we recommend researchers choose a compilation of best fit, continue to cite ERIC as seminal work on implementation strategies, and integrate both ISAC (or other compilations [27, 28]) and ERIC as appropriate. For example, the Food is Medicine (FIM) movement in the United States is a public health and clinical approach to improving food security, nutrition, and health among Americans with low income that occurs between community settings (e.g., food banks and other community-based organizations) and healthcare spaces [75]. Future work that integrates ERIC and ISAC may lead to suitable implementation strategies to advance FIM innovations that are fit to context.

In addition to selecting implementation strategies that fit context, selecting appropriate strategies relevant to implementation determinants is also needed. The results of this study reveal that most of the barriers interviewees addressed through implementation strategies were at the outer or inner setting level. However, previously reported common implementation strategies in community settings are training, educational materials, and outreach visits [14], which are designed to overcome barriers at the individual level (e.g., knowledge, skills) versus the inner or outer setting level. This indicates that commonly used implementation strategies reported in the literature may not be appropriate to address practice barriers. The ISAC compilation and strategy selection process could lead to greater use of relevant implementation strategies to address common barriers.

Given the established need for pragmatic implementation strategy selection guidance, we developed a four-step process informed by Leeman et al.’s streamlined approach [76], recommendations for accelerating the contextual inquiry process [77, 78], and our experiences selecting implementation strategies in community settings [44]. First, as recommended in Leeman et al.’s approach and other recent guidance on shortening the contextual inquiry process [76–78], we recommend reviewing available information on EBI integration, including both peer-reviewed (e.g., systematic reviews) and grey literature (e.g., practice reports) to identify multi-context implementation barriers and facilitators that have previously been captured. Then, a decision can be made on whether further contextual inquiry is needed to determine barriers and facilitators to EBI integration in the unique implementation setting. If further contextual inquiry is deemed necessary (e.g., there is minimal supporting evidence) to better understand implementation determinants, this could be done through formal research (e.g., using CFIR or other determinant frameworks [35, 71–74]). Alternatively, the process could be more pragmatic; for example, a team could complete a “premortem” session to uncover potential implementation outcomes (RE-AIM dimensions) to be addressed by implementation strategies [79, 80].

Second, respecting practitioners’ preference for a strength-based approach that includes both overcoming barriers and capitalizing on facilitators, we recommend working with partners to review practice materials and determine what implementation strategies are already in place – recognizing that they may not be referred to as implementation strategies (e.g., resources and supports) [81]. Third, in alignment with the contextual inquiry approach in step 1, we recommend using guidance tools (Tables 4 and 5) to select implementation strategies either by implementation outcomes (RE-AIM dimensions) or determinant framework levels. For example, a team may agree that strategies are primarily needed to influence adoption when introducing a new EBI. The team could use Table 4 to review strategies used to improve adoption, tailor potential strategies already in place, and come to consensus on additional strategies needed to integrate the EBIs. As another example, a review of the literature or contextual inquiry may identify that barriers to implementing built environment approaches to physical activity are primarily at the levels of the inner setting (e.g., relative priority, available resources) and the innovation (e.g., cost, complexity) [34, 82, 83]. Using a similar process, teams could review strategies indicated to alleviate inner setting and innovation barriers in Table 5 and select relevant strategies.

Fourth, we recommend teams tailor the selected strategies with health equity considerations in mind. Ideally, strategies should be selected at multiple levels to avoid focusing only on improving individual skills or knowledge without considering existing systems and structures. This includes considering the implementation strategies’ costs and the organization’s available resources, history, and other contextual factors [15, 20]. Next, the strategies should carefully be tailored based on cultural considerations and partner input to promote equity and avoid exacerbating disparities (e.g., by considering whether the strategy will be sufficient to improve adoption or implementation in settings with lower resources) [15, 20]. Finally, to promote “evolvability” (i.e., adaptations leading to sustainability and equitable impact) of EBIs and implementation strategies over time [20], teams are encouraged to consider from the onset how they will track modifications, such as through the Framework for Reporting Adaptations and Modifications-Expanded (FRAME) and the Framework for Reporting Adaptations and Modifications to Evidence-based Implementation Strategies (FRAME-IS) [84, 85].

The strategy selection process and ISAC compilation can be used by community setting researchers and practitioners to develop the evidence base regarding tailoring and testing ISAC strategies to determine what works to aid EBI adoption, implementation, and maintenance, for whom, and under what conditions. For this to occur, ISAC first needs to be disseminated to both researchers and practitioners. Results of the study inform how to disseminate ISAC to practitioners beyond peer-reviewed journal articles intended for research audiences. Trusted public health organizations’ role in sharing this compilation through webinars, listservs, and newsletters will be important. In general, the results of this study align with similar work regarding practitioners’ dissemination preferences; however, in prior investigations practitioners have preferred email and listserv communications [86–90]. The primary results reported in this study regarding the use of webinars may be an effect of increased virtual communication during and after the COVID-19 pandemic. In general, researchers value dissemination to practice audiences, but often are unsure of where to begin dissemination efforts [89, 90]. The results presented here provide suggestions for where to best place effort to reach practice audiences. Future research should test dissemination through specific sources and channels to determine how to best increase adoption of ISAC and associated tools by practitioner audiences.

Moving forward, the practical application of ISAC among research and practice settings should build on a few important considerations. First, as highlighted by this study, many researchers and practitioners already use implementation strategies to help overcome EBI barriers at multiple levels. However, gaps in terminology and alignment with implementation science theories, models, and frameworks have existed. ISAC may help to minimize this gap by providing standard terminology for implementation strategy application and outcomes regarding RE-AIM that could eventually be compared across community settings and EBIs. Comparing strategies that are already occurring may also help researchers and practitioners capture and report available assets or facilitators and advocate for funds to focus on overcoming barriers with more complex implementation strategies. Efforts to support community implementation researchers and practitioners to name, define, specify (e.g., the actor, action, target, timing, frequency, implementation outcome, and justification of strategies), track, and evaluate ISAC strategies over time in response to overcoming barriers of EBIs is needed to move the state of the science forward [9, 91].

As one final consideration, implementation outcomes are typically organizational-level RE-AIM dimensions: adoption, implementation, and maintenance. Reach has also been operationalized as an implementation outcome as there are implementation (and dissemination) strategies that can be used by researchers or practitioners to better reach the priority population [92, 93]. Effectiveness is not typically included as an outcome of implementation strategies (the “stuff” we do to help people and places do the “thing.”) [94]. However, we included effectiveness in this study and subsequent guidance tools because of the challenges associated with assessing effectiveness in real world settings on an ongoing basis, especially as programs are maintained long term with less academic or researcher control [20, 95]. In this vein, effectiveness may more accurately be considered as a proximal implementation outcome [8]. For example, improved program evaluation may lead to program maintenance through the documentation and sharing of results with funders and other invested parties [96, 97].

### Limitations

A robust approach to identifying implementation strategies used to overcome EBI barriers among community researchers and practitioners was used by an expert group who are active in the community implementation science field. However, this work is not without limitations. First, some of the interviewees had previous relationships with the research team. Given the topic of interviews was not overly sensitive, familiarity may have aided the conversation regarding ISAC. Given the rapid analysis method used, interview findings were not returned to participants to provide feedback, which may have enhanced interpretation.

Next, the qualitative approach used limited the number of participants who provided input on ISAC (e.g., compared to the 71 expert researchers who participated in the Delphi study to develop the ERIC compilation) [10]. We contend that, while our sample was smaller, our methods were sufficient to develop a comprehensive compilation and we were able to gather more rich, nuanced information than can be gleaned from surveys. Strengths and measures for achieving trustworthiness and transferability [67] include data collection from both researchers and practitioners, the RE-AIM-based interview guide that prompted for barriers and strategies used to overcome them, and the assessment of saturation. However, compiling the list of ISAC strategies and identifying the associated implementation determinants and outcomes was conducted as an initial step to moving forward the application and evaluation of implementation strategies in community settings. The initial ISAC compilation will likely be refined and updated over time, as other compilations have been, as more evidence is generated on strategy use across diverse settings (e.g., geographical regions, urban and rural areas) and EBIs [10, 98].

Lastly, related to the ISAC guidance tools, we opted to use broad domains instead of specific constructs (e.g., “inner setting” instead of “communications” or “culture”). As other scholars have found [49], it is difficult to identify links between specific determinants and specific implementation strategies, and single strategies are often linked to multiple barriers (or multiple strategies are needed to address a single barrier) [8]. As well, when frameworks or guidance tools become too granular, they do not apply to diverse settings [99]. Thus, using broad domains instead of specific constructs may be more useful for the barrier-strategy matching process.

## Conclusions

In summary, future steps to broadly disseminate the ISAC compilation so community researchers and practitioners will have a wider range of relevant strategies available to them are needed. Our hope is that others will use the ISAC compilation and label strategies by name to advance generalizable implementation science knowledge. Additional efforts to aid community researchers and practitioners in documenting the necessary components of ISAC strategy application may also be needed to build the evidence base on which ISAC strategies work, in which contexts, and for which implementation researchers and practitioners.

## Electronic supplementary material

Below is the link to the electronic supplementary material.

Supplementary Material 1

Supplementary Material 2

Supplementary Material 3

Supplementary Material 4

Supplementary Material 5

Supplementary Material 6

## Data Availability

The datasets analyzed during the current study are available from the corresponding author on reasonable request.

## Abbreviations

CCIS: Consortium for Cancer Implementation Science
CFIR: Consolidated Framework for Implementation Research
EBI: Evidence-based interventions
ERIC: Expert Recommendations for Implementing Change
FIM: Food is Medicine
ISAC: Implementation Strategies Applied in Communities
RE-AIM: Reach, Effectiveness, Adoption, Implementation, Maintenance
