# Supplementary material for: Advancing implementation science in community settings: the implementation strategies applied in communities (ISAC) compilation
Source: Int J Behav Nutr Phys Act. 2024 Nov 26;21:132. doi: 10.1186/s12966-024-01685-5 (PMC11590528; doi:10.1186/s12966-024-01685-5)
Supplement: Supplementary file 1 — Supplementary Material 1 [file 12966_2024_1685_MOESM1_ESM.docx]

Dear [name],

*For new contacts:* My name is Laura Balis, and I’m a researcher at Gretchen Swanson Center for Nutrition. [Referrer name] suggested I contact you OR I’m contacting you because of your experience with [experience area].

OR

*Personal greeting for previous contacts:* It was great to meet you at the [conference name]. OR I hope everything is going well at the [organization name].

*Researchers*: I’m working on an exciting new research study, with an end goal of creating a new compilation of implementation strategies for community settings. I’m reaching out to you today to invite (and encourage!) you to participate in a 45-minute Zoom interview to share your expertise and experience. We’ll ask about strategies you’ve used as you’ve worked to improve the adoption, implementation, and maintenance of evidence-based interventions (physical activity, nutrition, and/or tobacco prevention) in community settings. This research has been deemed exempt by the University of Nebraska Medical Center IRB, #0257-23-EX.

OR

*Practitioners:* I’m reaching out to you today to invite (and encourage!) you to participate in a 45-minute Zoom interview as part of a research study. The goal of the interview is to learn more about the strategies you use to improve the adoption, implementation, or maintenance of evidence-based interventions in your organization. The research study results will be used to develop a list of strategies to share with practitioners and researchers in community settings. This research has been deemed exempt by the University of Nebraska Medical Center IRB, #0257-23-EX.

If you are interested, please click here [insert screener link] to fill out a brief (five questions) screening form and schedule an interview. You will receive a $25 e-gift card for completing the interview, which you can use for a variety of retailers (e.g., Amazon, Target, grocery stores) or charity organizations.

Thank you!

Take care,

Laura

Laura Balis, PhD

Research Scientist

Gretchen Swanson Center for Nutrition

14301 FNB Parkway, Suite 100, Omaha, NE 68154

Direct: (531) 895-4138

Remote Location: St. George, UT (Mountain Time)

Follow-up email:

Hi [name],

I’m sending a quick note to follow up on my previous email about participating in a 45-minute Zoom interview as part of a research study. The goal of the interview is to learn more about the strategies you use to improve the adoption, implementation, or maintenance of evidence-based interventions in your organization. This research has been deemed exempt by the University of Nebraska Medical Center IRB, #0257-23-EX.

If you are interested, please click here [insert screener link] to fill out a brief (five questions) screening form and schedule an interview. You will receive a $25 e-gift card for completing the interview, which you can use for a variety of retailers (e.g., Amazon, Target, grocery stores) or charity organizations.

Thank you!

Laura

Laura Balis, PhD

Research Scientist

Gretchen Swanson Center for Nutrition

14301 FNB Parkway, Suite 100, Omaha, NE 68154

Direct: (531) 895-4138

Remote Location: St. George, UT (Mountain Time)
