# Supplementary material for: Advancing implementation science in community settings: the implementation strategies applied in communities (ISAC) compilation
Source: Int J Behav Nutr Phys Act. 2024 Nov 26;21:132. doi: 10.1186/s12966-024-01685-5 (PMC11590528; doi:10.1186/s12966-024-01685-5)
Supplement: Supplementary file 2 — Supplementary Material 2 [file 12966_2024_1685_MOESM2_ESM.docx]

**Eligibility Screener – Researchers**

Thank you for your willingness to complete this screening survey and schedule an interview. We will protect the information you provide by safely storing your responses. You can choose to not answer any questions you do not want to answer or to not complete the survey. If you complete this survey, it will be included in a research study to develop a compilation of implementation strategies for community settings. This research has been deemed exempt by the University of Nebraska Medical Center IRB, #0257-23-EX.

1. Please provide your first and last name [open-ended]

2. In which setting(s) have you conducted community-engaged research? [select all that apply]

- Education
- Social services
- City planning and transportation
- Workplaces
- Recreation/sport
- Faith-based
- Other public health settings
  - *If other:* Please describe. [Open-ended]

3. How many years of experience do you have conducting community-engaged research in these settings? [open-ended]

4. How would you rate your level of expertise in implementation science?

- Beginner
- Intermediate
- Advanced

5. Through your community-engaged research, have you worked to improve the adoption, implementation, or maintenance of an evidence-based intervention aimed at improving physical activity, nutrition, or tobacco patterns/practices?

- Yes
- No

*If no, go to thank you message and end survey.*

*If yes:*

6. What are the evidence-based interventions? (Please list up to three interventions) [open-ended]

*For each intervention listed:*

What level of influence is the intervention? [select all that apply]

- Individual or interpersonal
- Policy, systems, or environment

What are the primary outcomes of the intervention? [select all that apply]

- Physical activity
- Nutrition
- Tobacco use

Thank you for completing this survey. We will review your responses and follow up with eligible respondents to schedule an interview.
