# Supplementary material for: Advancing implementation science in community settings: the implementation strategies applied in communities (ISAC) compilation
Source: Int J Behav Nutr Phys Act. 2024 Nov 26;21:132. doi: 10.1186/s12966-024-01685-5 (PMC11590528; doi:10.1186/s12966-024-01685-5)
Supplement: Supplementary file 3 — Supplementary Material 3 [file 12966_2024_1685_MOESM3_ESM.docx]

**Eligibility Screener – Practitioners**

Thank you for your willingness to complete this screening survey and schedule an interview. We will protect the information you provide by safely storing your responses. You can choose to not answer any questions you do not want to answer or to not complete the survey. If you complete this survey, it will be included in a research study to develop a list of strategies for researchers and practitioners in community settings. This research has been deemed exempt by the University of Nebraska Medical Center IRB, #0257-23-EX.

1. Please provide your first and last name. [Open-ended]

2. In which setting(s) do you work? [select all that apply]

- Education
- Social services
- City planning and transportation
- Workplaces
- Recreation/sport
- Faith-based
- Other public health
  - *If other:* Please describe. [Open-ended]

3. How many years of experience do you have working in public health / prevention in these settings? [open-ended]

4. Do you manage or coordinate evidence-based programs delivered by staff or volunteers aimed at improving physical activity, nutrition, or tobacco patterns/practices?

- Yes
- No

*If no , go to thank you message and end survey.*

*If yes:*

5. Which evidence-based programs have you implemented? (Please describe up to 3 examples) [open-ended]

*For each intervention listed:*

What level of influence is the intervention? [select all that apply]

- Individual or interpersonal
- Policy, systems, or environment

What are the primary outcomes of the intervention? [select all that apply]

- Physical activity
- Nutrition
- Tobacco use

Thank you for completing this survey. We will review your responses and follow up with eligible respondents to schedule an interview.
