# Supplementary material for: Advancing implementation science in community settings: the implementation strategies applied in communities (ISAC) compilation
Source: Int J Behav Nutr Phys Act. 2024 Nov 26;21:132. doi: 10.1186/s12966-024-01685-5 (PMC11590528; doi:10.1186/s12966-024-01685-5)
Supplement: Supplementary file 5 — Supplementary Material 5 [file 12966_2024_1685_MOESM5_ESM.docx]

**Semi-Structured Interview Guide: Practitioners**

Thank you for making time for the interview today. I appreciate it! The goal of the interview is to learn more about the strategies you use to improve the adoption, implementation, or maintenance of evidence-based interventions in your organization. These strategies are typically used at the staff or setting level to support the people who deliver the program.

I’ll be asking you about evidence-based programs you’ve been involved with - these could be direct education programs or at the policy, systems, or environment level -, barriers or facilitators you experienced in integrating them, and what implementation strategies you used.

This study has been identified as exempt research by the University of Nebraska IRB. All reporting based on interviews for this research will be done in aggregate, with a goal of describing the main themes that emerge. Some quotes may be used, but we will never use names or identifiable information as part of these quotes.

I want to assure you that any answers and information you provide will be kept confidential. Feel free to speak openly, but also know that you do not have to answer any questions you feel uncomfortable responding to, and you may end the interview at any time and for any reason.

Do you have any questions about that or about the study overall?

It is okay if I audio-record this interview? **IF YES, START ZOOM RECORDING NOW**

I see that you have implemented [insert names of evidence-based interventions from screener survey]. **Can you briefly describe each of those programs or interventions?**

1. What challenges have you experienced in getting staff or delivery settings to agree to deliver these interventions? *[Adoption]*
   - **Prompts:** Determining who should deliver the intervention? Tracking adoption rates? Enhancing adoption in low-resources settings?
   - What strategies did you use to overcome these barriers?
2. Did you experience challenges in ensuring that these interventions were delivered with fidelity to the core components (i.e., were delivered as intended)? What about encouraging minor changes to tailor the program for the priority population? *[Implementation: fidelity]*
   - **Prompts:** Tracking adjustments and adaptations? Improving performance to increase fidelity?
   - What strategies did you use to overcome these barriers?
3. What about challenges with the resources required to deliver these interventions? *[Implementation: cost]*
   - **Prompts:** Time? Personnel? Equipment? Other financial costs?
   - What strategies did you use to overcome these barriers?
4. Once these interventions were implemented, did you have any challenges in recruiting the priority population? [*Reach*]
   - **Prompts:** Helping priority population overcome barriers to participating? Recruiting those who experience health disparities? Planning, advertising, promoting the intervention? Engaging community partners to help with recruitment?
   - What strategies did you use to overcome these barriers?
5. Also, once the interventions were underway, were there any challenges with evaluation? *[Effectiveness]*
   - **Prompts:** Determining the key changes/outcomes? Collecting data?
   - What strategies did you use to overcome these barriers?
6. Were you able to measure long-term impacts on community members’ health? What made it difficult? *[Maintenance: individual]*
   - **Prompts:** Staying in touch with participants after the intervention ends? Follow-up evaluation methods?
   - What strategies did you use to overcome these barriers?
7. Finally, thinking about sustainability, were you able to continue delivering these interventions after the initial implementation period? What made it difficult? *[Maintenance: organizational]*
   - **Prompts:** Plans for leaving resources or trained staff in place? Tools or resources for long-term monitoring and adaptation?
   - What strategies did you use to overcome these barriers?
8. Are there any other strategies you use to improve the adoption, implementation, or maintenance of evidence-based interventions in your organization that we haven’t discussed yet?
   - When did you use them? What barriers did you overcome?
9. Can we contact to you participate in a follow-up survey to help us refine the list of strategies we learn about through interviews?
10. Finally, the goal of this research is to develop a resource of implementation strategies for practitioners in community settings. How would you most likely learn about this type of resource?
    - **Prompts:** reports, meetings, presentations, conferences, websites, email
    - Who would typically share this type of information with you?
    - **Prompts:** colleagues, supervisors, outside organizations
